# PbEIL1 acts upstream of *PbCysp1* to regulate ovule senescence in seedless pear

**DOI:** 10.1038/s41438-021-00491-5

**Published:** 2021-03-10

**Authors:** Huibin Wang, Haiqi Zhang, Fangfang Liang, Liu Cong, Linyan Song, Xieyu Li, Rui Zhai, Chengquan Yang, Zhigang Wang, Fengwang Ma, Lingfei Xu

**Affiliations:** College of Horticulture, Northwest A&F University, Yangling, Shaanxi Province China

**Keywords:** Developmental biology, Non-model organisms

## Abstract

Numerous environmental and endogenous signals control the highly orchestrated and intricate process of plant senescence. Ethylene, a well-known inducer of senescence, has long been considered a key endogenous regulator of leaf and flower senescence, but the molecular mechanism of ethylene-induced ovule senescence has not yet been elucidated. In this study, we found that blockage of fertilization caused ovule abortion in the pear cultivar ‘1913’. According to transcriptome and phytohormone content data, ethylene biosynthesis was activated by pollination. At the same time, ethylene overaccumulated in ovules, where cells were sensitive to ethylene signals in the absence of fertilization. We identified a transcription factor in the ethylene signal response, ethylene-insensitive 3-like (EIL1), as a likely participant in ovule senescence. Overexpression of *PbEIL1* in tomato caused precocious onset of ovule senescence. We further found that EIL1 could directly bind to the promoter of the *SENESCENCE-ASSOCIATED CYSTEINE PROTEINASE 1* (*PbCysp1*) gene and act upstream of senescence. Yeast one-hybrid and dual-luciferase assays revealed the interaction of the transcription factor and the promoter DNA sequence and demonstrated that PbEIL1 enhanced the action of *PbCysp1*. Collectively, our results provide new insights into how ethylene promotes the progression of unfertilized ovule senescence.

## Introduction

Seedlessness, a very desirable trait that improves ease of consumption, has been bred into many fruit species, including grape^1^, litchi^2^, citrus^3^, and watermelon^4^. Previous studies have reported that phytohormones (e.g., gibberellins [GAs], auxin, and cytokinins), fertilization failure, female and male sterility, embryo abortion, and various other factors can induce the formation of seedlessness^4–9^.

In angiosperms, pollination and fertilization are necessary for ovule and fruit development^10^. After pollination, the pollen tube enters the embryo sac and releases two sperm cells. One sperm cell fuses with the egg cell to form a diploid embryo, while the other sperm cell fuses with the two polar nuclei to form a triploid endosperm. A normal seed is composed of three genetically distinct tissues: the embryo, endosperm, and seed coat^11,12^. The seed coat, including the inner and outer integuments, is necessary for ovule development. Multiple pathways are involved in ovule and fruit development after pollination, and a number of independent and possibly redundant hormone pathways regulate development at the early process^13^. In orchids, ovary and gametophyte development are coordinately regulated by auxin and ethylene after pollination. In the presence of auxin, ethylene regulates the initiation of ovary development and indirectly promotes subsequent ovule differentiation^14^. In *Arabidopsis thaliana*, GA regulates integument development by interfering with the transcription factor ABERRANT TESTA SHAPE^15^. In tomato, auxin and GA rapidly accumulate after pollination and act as positive regulatory signals during early fruit development^16^.

As a key hormone, ethylene regulates many aspects of the plant life cycle, such as fertilization, seed germination, flower development, sex determination, senescence, fruit ripening, and responses to biotic and abiotic stresses^17^. In early maize development, ethylene mediates programmed cell death (PCD) during endosperm development and regulates carpel senescence^18,19^. Ethylene also regulates carpel senescence in *Pisum sativum*^19^. Furthermore, ethylene controls both petal and unfertilized whole-pistil senescence^20^. In the ethylene biosynthesis and signal transduction pathways, methionine is first converted to S-AdoMet by S-AdoMet synthetase. S-AdoMet is then transformed into 1-aminocyclopropane-1-carboxylic acid (ACC) by ACC synthase (ACS). Under the catalytic action of ACC oxidase (ACO), ACC is finally converted to ethylene. The formation of ACC is the rate-limiting step in this pathway^17^. In ethylene signal transduction, ethylene-insensitive 3 (Ein3) and Ein3-like 1 (EIL1) control most ethylene responses and regulate the vast majority of downstream target genes^21–24^. Previous studies have reported that ethylene plays a critical role in preventing the attraction of a second pollen tube and that activation of the Ein3-dependent ethylene response pathway is necessary for pollen tube attraction^25^. Furthermore, overaccumulation of *Ein3* in the micropyle blocks pollen tube attraction^26^. Ein3 also regulates ovule senescence to modulate GA-mediated fruit set in Arabidopsis^27^. Although these findings highlight the role of ethylene in early ovule development and the involvement of this hormone in ovule senescence, little is known about the mechanism by which ethylene controls ovule senescence.

In plants, PCD occurs during: sex determination; anther development; tracheary element differentiation; degeneration of the nucellus, endosperm, and endothelium; leaf, carpel, and petal senescence; and early plant senescence^18,19,27–33^. In plants, most enzymes in the large cysteine protease (Cysp) family belong to the papain-like, metacaspase, and legumain subfamilies^34^. Cysp enzymes are ubiquitously involved in cell degeneration and senescence in plants and are induced during processes such as the differentiation of tracheary elements^35^. In Arabidopsis, *senescence-associated gene 12 (SAG12)*, *SAG2*, *XYLEM CYSTEINE PROTEASE 1* (*XCP1*), and *XCP2* are closely connected to cell senescence^36–38^. *AtSAG12* is a senescence marker gene induced during leaf senescence^36,39^. In *Brassica napus*, BnCysp1 is an ovule integument-specific cysteine proteinase associated with PCD of the inner integument^33^. Despite these findings, a relationship between ethylene and *Cysp* genes has not been reported.

In this study, we aimed to characterize the involvement of ethylene in the initiation and progression of ovule senescence in unfertilized pear. To achieve this goal, we analyzed the expression patterns and functions of ethylene-related genes as well as senescence-related target genes acting downstream of the ethylene signal. Our analysis revealed that the PbEIL1-dependent regulation of PbCysp1 controls the senescence of ovules experiencing fertilization blockage.

## Results

### Fertilization blockage leads to ovule abortion in the seedless pear cultivar ‘1913’

**Fig. 1:**
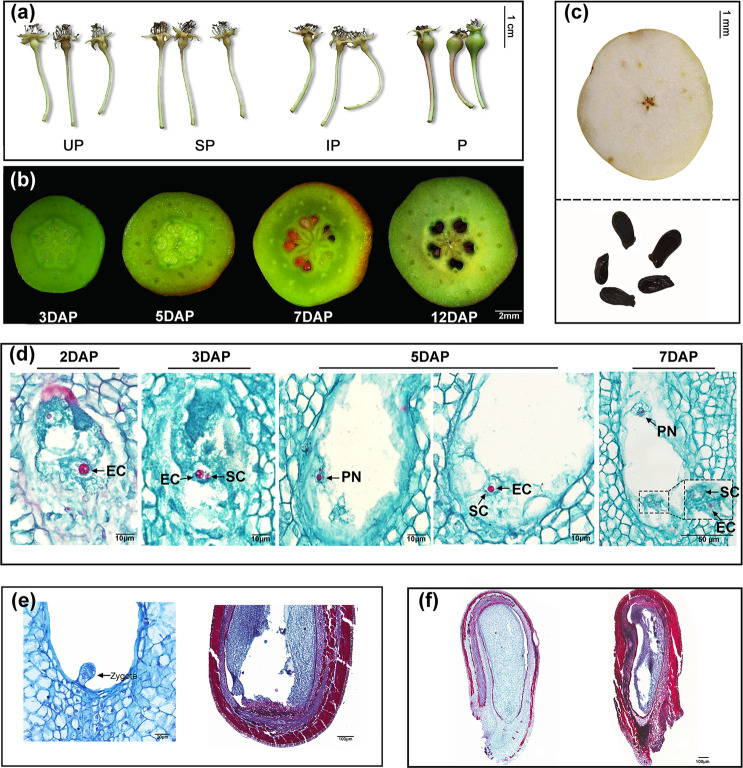
Morphological and histopathological characteristics of different tissues of ‘1913’ and ‘Dangshansu’ pear. **a** Developing ‘1913’ fruitlets subjected to different treatments and then observed at 12 days after treatment. UP, unpollinated; SP, self-pollinated; IP, pollinated with inactivated pollen; P, cross-pollinated. **b**, **c** Development of ‘1913’ ovules and fruits at early (**b**) and mature (**c**) stages. At 7 DAP, the ovules began to abort. **d** Paraffin section-based observation of the fertilization process in ‘1913’ prior to ovule death. EC, egg cell; SC, sperm cell; PN, polar nucleus. **e** Embryo sac and **f** ovule development at 12 DAP. In both images, ‘Dangshansu’ and ‘1913’ are on the left and right, respectively. A zygotic embryo was formed in ‘Dangshansu’, whereas the embryo sac of ‘1913’ was empty. The different tissues were stained with safranin and fast green for histocytological observation. DAP, days after pollination

To verify the cause of seedlessness in ‘1913’ pear, we carried out field experiments, including artificial emasculation followed by bagging (nonpollination), self-pollination with bagging, artificial pollination of inactivated pollen followed by bagging, and cross-pollination. According to our results, fruit development and set occurred 12 days after pollination (DAP) in pear subjected to cross-pollination, whereas the fruit of the pear plants subjected to the other treatments fell off (Fig. 1a). Cross-pollination is thus necessary for fruit set in ‘1913’ pear. We next monitored the development of early ovules subjected to cross-pollination treatment. We observed that the ovules began to turn brown at 7 DAP and were completely brown at 12 DAP (Fig. 1b). In addition, the seeds in mature fruits were shriveled (Fig. 1c). When we stained ovules with Alcian blue and nuclear fast red after pollination, we found that most integument cells of ‘1913’ were stained deeply blue at 7 DAP, and a notable amount of intercellular space was observed in the integument area (Supplementary Fig. S1). In contrast, ovule cells of the seeded variety ‘Dangshansu’, used as a control, remained intact and were barely stained blue (Supplementary Fig. S1). These results demonstrate that integument cells of ‘1913’ begin to die and degenerate at 7 DAP.

To determine the reason for ovule abortion, we collected ovules of ‘1913’ every 24 h after pollination to check for ovule abortion. We found that sperm cells entered the embryo sac at 3 DAP but did not fuse with the egg cell or polar nucleus cells until 7 DAP—when the ovule began to abort (Fig. 1b, d). At 12 DAP, an empty embryo sac was present, and the ovule cells had died (Fig. 1e, f). In ‘Dangshansu’, in contrast, a developing zygotic embryo formed, and ovule cells remained intact (Fig. 1e, f). All these results indicate that fertilization failure is the fundamental cause of ovule abortion in ‘1913’.

### Participation of **e**thylene in the abortion of unfertilized ovules in ‘1913’ pear

To determine the mechanism of ovule abortion in ‘1913’, we collected ovules of ‘1913’ and ‘Dangshansu’ at 0, 3, and 7 DAP for transcriptome analysis. To verify the RNA-seq results, quantitative real-time PCR (qRT-PCR) was conducted on candidate genes. The results of the qRT-PCR analysis suggest that the RNA-seq data from this study are reliable (Supplementary Fig. S2). Venn diagrams were constructed to visualize the distributions of genes differentially expressed between ‘1913’ and ‘Dangshansu’ during the three ovule developmental periods (0, 3, and 7 DAP) (Supplementary Fig. S3a). Overlapping genes in these comparisons were used to analyze the molecular mechanism underlying ovule development after pollination. A Kyoto Encyclopedia of Genes and Genomes (KEGG) enrichment analysis indicated that differentially expressed genes (DEGs) were significantly enriched in 20 pathways, especially those related to biosynthesis of amino acids, carbon metabolism, and plant hormone signal transduction (Supplementary Fig. S3b and Supplementary Table S2). These analyses revealed that multiple physiological mechanisms are involved in ovule development after pollination and fertilization. In subsequent analyses, we focused on significantly enriched DEGs involved in plant hormone signal transduction.

**Fig. 2:**
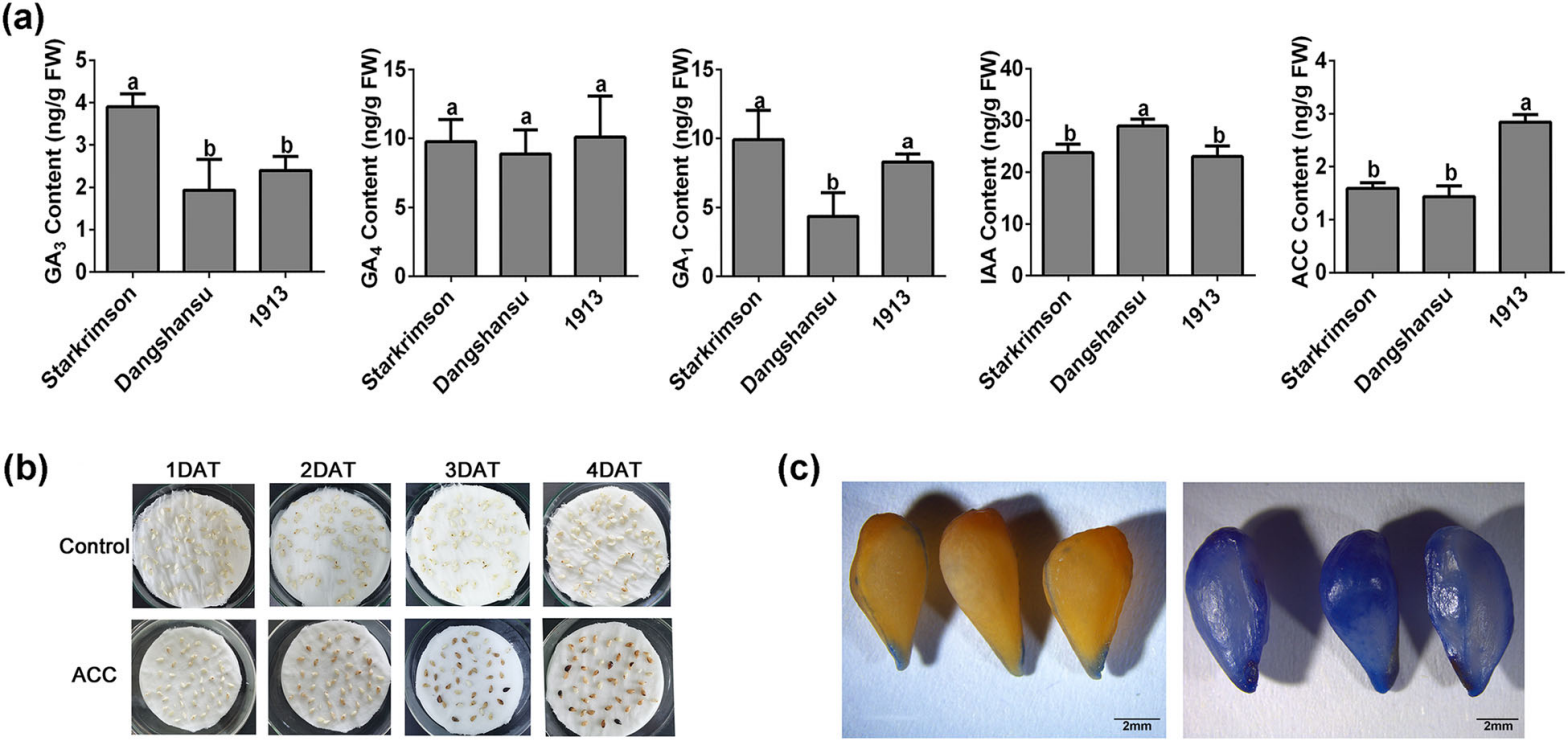
Involvement of ethylene in ovule abortion in ‘1913’. **a** Levels of active gibberellins (GA_1_, GA_3_, and GA_4_), IAA, and ACC in the ovules of ‘1913’ and the seeded cultivars ‘Dangshansu’ and ‘Starkrimson’. **b** Observations of ovule development in vitro after different treatments. ACC, exogenous ACC treatment; Control, solvent treatment. **c** Ovule senescence was observed by trypan blue staining. Control and ACC-treated ovules are on the right and left, respectively. DAT, days after treatment

Because heatmaps revealed that pollination activated auxin, GA, and ethylene biosynthesis and signal transduction pathways (Supplementary Fig. S3c and Supplementary Table S3), we investigated whether ovule abortion was due to significant differences in hormone content between seeded and seedless varieties. We thus compared active GA, auxin, and ACC levels among ‘1913’ and two-seeded varieties, ‘Dangshansu’ and ‘Starkrimson’. We found that ACC, the precursor of ethylene and thus a marker of endogenous ethylene content, was the only hormone whose content was significantly different in ‘1913’ vs. the two-seeded varieties. This result suggests that ethylene is related to the death of unfertilized ovules. To verify this inference, we designed an experiment in which normal ovules were treated with exogenous ACC in vitro. In this experiment, most ACC-treated ovules, but few control ovules, turned dark at 4 days after treatment, thus implying that ACC may promote ovule senescence in vitro (Fig. 2b). Trypan blue staining revealed that ACC treatment resulted in premature ovule cell death. Taken together, these results indicate that ethylene participates in the degradation of abnormal ovules of ‘1913’. In our next analysis, we focused on how ethylene contributes to unfertilized ovule death.

### Role of PbEIL1 in unfertilized ovule senescence

**Fig. 3:**
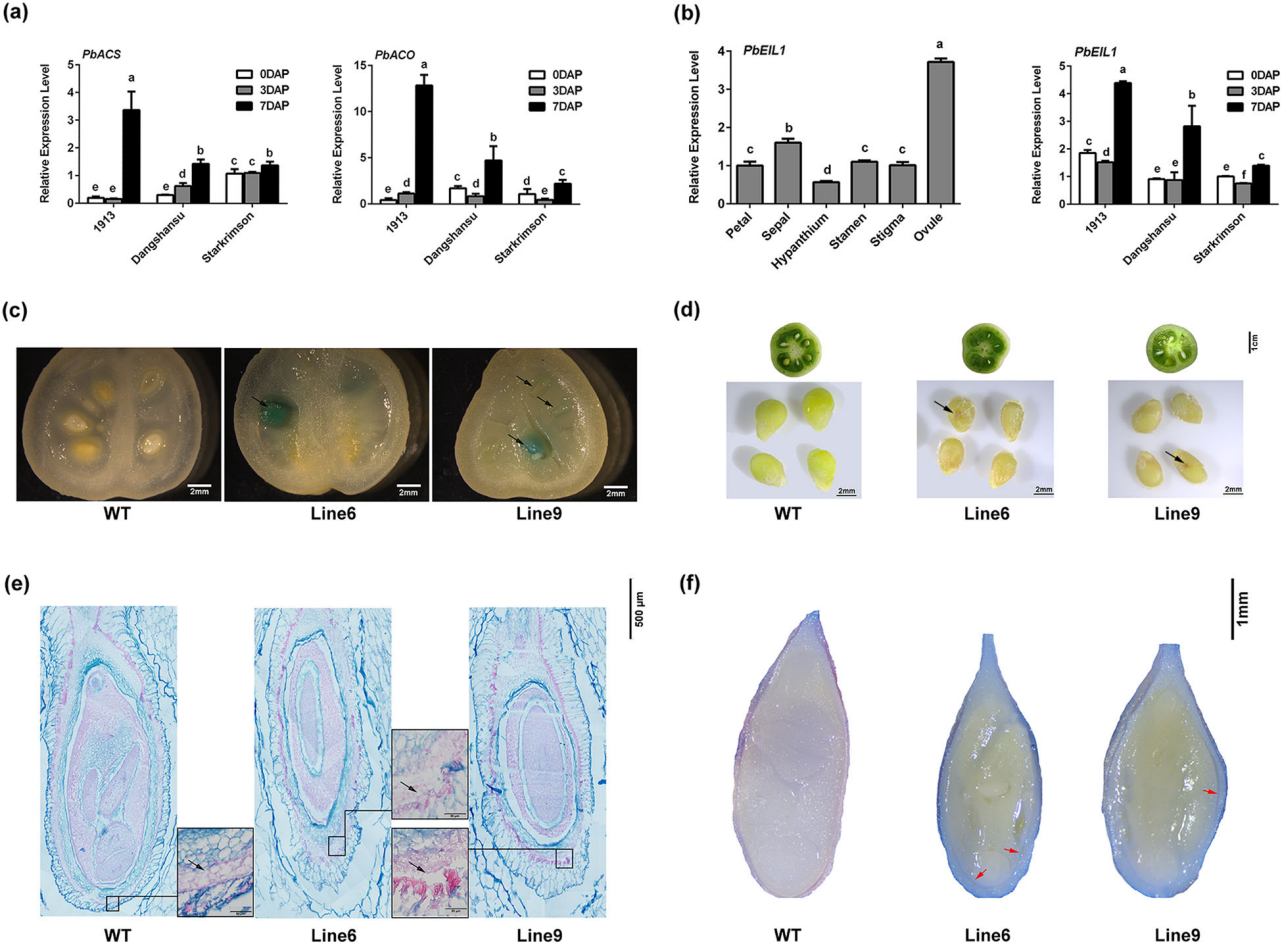
Acceleration of seed senescence in *PbEIL1*-overexpressing tomato. **a** *PbACS* and *PbACO* expression patterns at different stages in seeded pear cultivars and ‘1913’. **b** Tissue-specific and spatiotemporal expression of *PbEIL1*. **c** Identification of positive transgenic plants by GUS staining. The black arrows indicate the longitudinal sections of the ovules. The staining revealed the expression of *PbEIL1* under the control of the PeFBP7 promoter in the ovule integument. **d** Seed development of 20-day-old fruits. The black arrows point to areas of significant seed senescence. **e** Paraffin sections of developing integument cells stained with Alcian blue and nuclear fast red. The black arrows indicate regions where the structures of transgenic and WT cells were significantly different. **f** Observation of seed death by trypan blue staining. The inner integuments of transgenic seeds were obviously stained (red arrows). In (**a**) and (**b**), the expression levels of each gene in ‘Starkrimson’ at 0 DAP and the expression of *PbEIL1* in petals were normalized to 1.0. The data are mean ± SDs of three biological replicates. Different letters between bars indicate significant differences at *P* < 0.05 (Duncan’s multiple range test)

In this portion of the study, we first examined the expression patterns of ethylene synthesis-related genes after pollination in the ovules of seedless and seeded pear. We collected ovules of ‘1913’, ‘Dangshansu’, and ‘Starkrimson’ at 0, 3, and 7 DAP for analysis. We found that *PbACS* and *PbACO* family genes were significantly upregulated in ‘1913’, ‘Dangshansu’, and ‘Starkrimson’ at 7 DAP compared with 0 and 3 DAP. At 7 DAP, the two classes of genes were significantly more upregulated in seedless pear than in seeded pear (Fig. 3a and Supplementary Fig. S4). These results demonstrate that pollination activates ethylene biosynthesis and that ethylene is synthesized at higher levels in seedless pear than in seeded pear. Ein3, a transcription factor that can activate ethylene responses, may play a key role in the relationship between ethylene overaccumulation and ovule abortion. Taking into account that *AtEin3* is expressed in ovules at the early stage in Arabidopsis^26^, we searched for the closest homolog of *AtEin3* in pear and found it to be *PbEIL1* (61% identity). Analysis of integrated transcriptomic data indicated that *PbEIL1* was upregulated at 7 DAP in ‘1913’ (Supplementary Table S3). In light of these results, we used *PbEIL1* to further ascertain the relationship between ethylene signaling and senescence.

We analyzed the tissue expression specificity of *PbEIL1* and found that *PbEIL1* was more highly expressed in ovules than in petals, sepals, hypanthia, stamens, and stigmas (Fig. 3b). In a qRT-PCR assay, *PbEIL1* was significantly upregulated at 7 DAP in ‘1913’, ‘Dangshansu’, and ‘Starkrimson’, with the highest expression observed in ‘1913’ (Fig. 3b). These results suggest that *PbEIL1* is associated with early ovule development and indicate that the high level of *PbEIL1* in ‘1913’ unfertilized ovules may be involved in the senescence of abnormal ovules.

To verify the relationship between *PbEIL1* and unfertilized ovule abortion, we expressed *PbEIL1* in tomato under the control of the ovule-specific *PeFBP7* promoter. By monitoring GUS signals, we found that *PbEIL1* was expressed in the ovule integument of transgenic tomato (Line 6 and Line 9) (Fig. 3c). When we observed the developing seeds at 20 DAP, we found that the transgenic seeds were yellower than wild-type seeds and had some black areas (Fig. 3d). This finding indicates that *PbEIL1* accelerates seed senescence. Observation of paraffin sections and trypan blue staining confirmed the senescence of the transgenic seeds (Fig. 3e, f). The structures of inner integument cells were incomplete in transgenic seeds (Fig. 3e), and their integument was strongly stained by trypan blue as a result of senescence. These results demonstrate that *PbEIL1* participates in the senescence process of unfertilized ovules in ‘1913’.

### Screening of *BnCysp1* homologs in pear

**Fig. 4:**
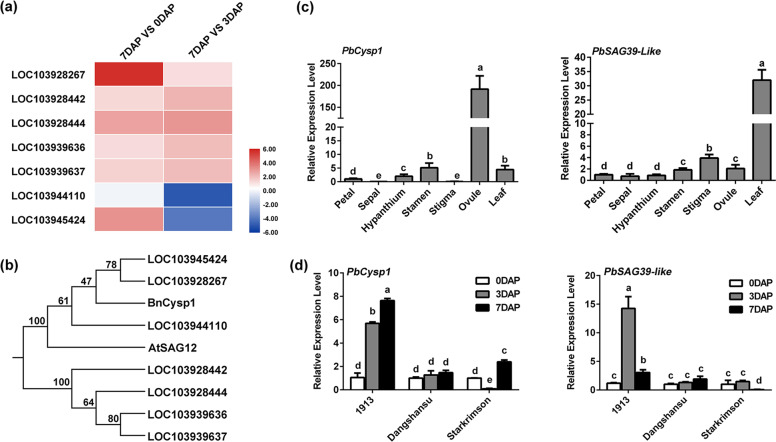
Expression patterns of *PbCysp1* and *PbSAG39-like*. **a** Heatmap showing the differential expression of all *PbCysp* genes in the ‘1913’ transcriptome. **b** Phylogenetic tree of amino acid sequences of Cysp proteins in pear and senescence-related Cysp proteins in *Arabidopsis thaliana* and *Brassica napus*. **c** Tissue-specific expression of *PbCysp1* and *PbSAG39-like*. **d** *PbCysp1* and *PbSAG39-like* expression patterns at different stages in ‘1913’, ‘Dangshansu’, and ‘Starkrimson’. The expression levels of each gene in ‘Starkrimson’ at 0 DAP and those of *PbCysp1* and *PbSAG39-like* in petals were normalized to 1.0. The data are mean ± SDs of three biological replicates. Different letters between bars indicate significant differences at *P* < 0.05 (Duncan’s multiple range test)

In angiosperms, the integument is an important structure in ovule development. In ‘1913’, ovule abortion was associated with apparent death of the integument (Fig. 1e, f). Previous studies have shown that *BnCysp1* and *ATSAG12* specifically regulate integument and leaf death, respectively, and that the expression levels of these genes are elevated when the corresponding tissues undergo senescence^33,40^. These findings implicate senescence-specific cysteine proteases in the senescence of different tissues. In light of these results, we analyzed transcriptomic data for ‘1913’ to identify senescence-specific DEGs possibly involved in ovule death. A heatmap visualizing differences in the expression levels of these genes is shown in Fig. 4a. An evolutionary analysis of homologous proteins indicated that PbCysp1 (LOC103928267) and PbSAG39-like (LOC103945424) were more closely related to BnCysp1 than to ATSAG12 (Fig. 4b). We collected different floral organs at 7 DAP and mature leaves at 153 DAP and performed a qRT-PCR assay, which revealed that *PbCysp1* was highly expressed in ovules but only weakly expressed in mature leaves and other floral organs. The expression of *PbSAG39-like* was highest in leaves, with lower expression in stigmas, ovules, and stamens (Fig. 4c). *PbCysp1* and *PbSAG39-like* were significantly upregulated at 3 DAP and 7 DAP compared with 0 DAP in ‘1913’, whereas no expression changes were detected in ‘Dangshansu’. In ‘Starkrimson’, however, *PbCysp1* was upregulated and *PbSAG39-like* was downregulated at 7 DAP relative to 0 DAP and 3 DAP, respectively (Fig. 4d). These results suggest that *PbCysp1* is an ovule-specific senescence-related gene, and that *PbSAG39-like* is primarily associated with leaf senescence. Consequently, *PbCysp1* is more similar to *BnCysp1* than *PbSAG39-like*, and the accumulation of *PbCysp1* is related to ovule abortion in ‘1913’. We thus selected *PbCysp1* for further study.

### Direct binding of PbEIL1 to the promoter of *PbCysp1* leads to ovule senescence

**Fig. 5:**
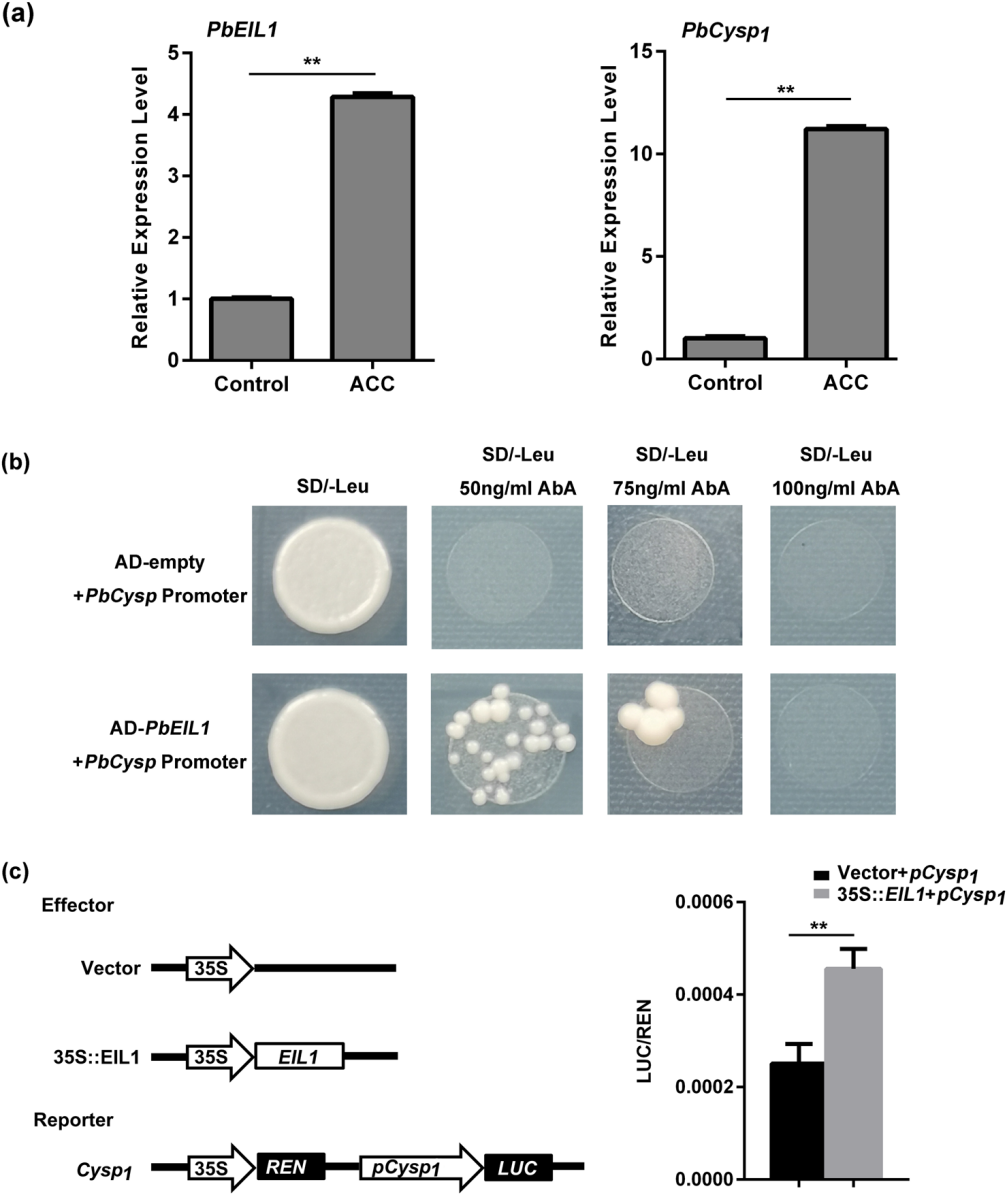
PbEIL1 promotion of *PbCysp1* expression by direct binding to the *PbCysp1* promoter. **a** Promotion of *PbEIL1* and *PbCysp1* expression by ACC treatment. **b** Yeast one-hybrid assay results showing the interaction of PbEIL1 with the *PbCysp1* promoter. **c** Validation of the activation effect of PbEIL1 on the *PbCysp1* promoter based on a dual-luciferase assay. Left: schematic diagrams of effector and reporter constructs used in the transient dual-luciferase assay; right: image revealing that LUC relative activity was significantly increased when *PbEIL1* was overexpressed. Relative promoter activity is represented by the ratio of the activity of the structural gene luciferase (LUC) to that of 35S Renilla (REN). An empty vector was used as a reference. Asterisks indicate significantly elevated LUC activity compared with that in the negative control (^**^P < 0.01, Student’s *t*-test)

According to previous studies, Ein3 family members can bind to the DNA sequence A(C/T)G(A/T)A(C/T)CT^41,42^. We analyzed the *PbCysp1* promoter sequence and found Ein3 binding sites. In addition, we observed that the phenotype of *PbEIL1*-transgenic tomato was similar to a phenotype in *B. napus* attributed to *BnCysp*. Taking all of these results into consideration, we hypothesized that *PbEIL1* and *PbCysp1* coregulate the death of ‘1913’ ovules. When we compared the expression patterns of *PbEIL1* and *PbCysp1*, we found that both genes were highly expressed in ovules and significantly upregulated at 7 DAP in ‘1913’ (Fig. 3b–d). In addition, *PbEIL1* and *PbCysp1* were both upregulated in ACC-treated ovules (Fig. 5a). In general, *PbEIL1* and *PbCysp1* had similar expression patterns and were regulated by ethylene. In a yeast one-hybrid assay, PbEIL1 was able to bind to the *PbCysp1* promoter (Fig. 5b). Moreover, dual-luciferase assays revealed that PbEIL1 could promote the activity of *PbCysp1* (Fig. 5c). These results demonstrate that PbEIL1 is upstream of *PbCysp1* and positively regulates the expression of *PbCysp1*. Collectively, our observations indicate that overaccumulation of PbEIL1 in ovules activates *PbCysp1*, resulting in ovule senescence.

## Discussion

In this study, we identified and characterized a previously unknown molecular interaction between the ethylene pathway and ovule senescence, a discovery that enhances our understanding of the gene regulatory networks governing ovule abortion. In summary, we have demonstrated that PbEIL1 binds to the promoter of *PbCysp1* to participate in unfertilized ovule abortion at the early stage. Previous studies have reported that auxin, GA, and ethylene increase in ovules after pollination and participate in the regulation of early ovule development^17,43–45^. In the present study, we similarly found that auxin, GA, and ethylene biosynthesis-associated genes are activated after pollination in pear ovules, with significant increases observed at 7 DAP (Fig. 3a and Supplementary Figs. S1, S4). When we compared the levels of these hormones between the seedless pear cultivar ‘1913’ and the seeded pear cultivars ‘Dangshansu’ and ‘Starkrimson’, we found that ACC was the only hormone whose content was significantly higher in seedless than in seeded pear; no consistent differences in auxin and GA levels were observed among the three varieties (Fig. 2a). This result thus suggests a prominent role for ethylene in ovule abortion in ‘1913’ and indicates that auxin and GA do not play key roles in this process. In this study, the selection of two different pear varieties as controls helped us to exclude the influences of genetic background differences. Nevertheless, our interpretations using data derived from different cultivars should be treated with some degree of caution.

In a previous study, Carbonell-Bejerano et al.^45^ reported that ethylene biosynthesis and response genes were activated upon unfertilized ovule senescence, a finding similar to our observations that ethylene is associated with ovule senescence. In our in vitro assay, spraying with ACC accelerated the senescence of pollinated ovules of ‘Dangshansu’ (Fig. 2b), which further verified the involvement of ethylene in ovule cell senescence. In this in vitro experiment, ovules at 30 DAP were more useful than early ovules for drawing conclusions because they were more resistant to mechanical damage inflicted by the experiment.

In the ethylene signaling pathway, *Ein3* and *EIL1* regulate the vast majority of downstream target genes^22–24^. Although ethylene is involved in the senescence of many different tissues in plants, most studies examining the role of ethylene in senescence have focused on leaves and petals^22–24,46^. Ethylene is known to participate in ovule senescence^27^, but no direct evidence exists to prove how ethylene causes ovule death. In our study, *PbEIL1* was significantly increased in the ovules of ‘1913’ at 7 DAP, when the ovules began to abort (Fig. 3b). Ectopic expression of *PbEIL1* in tomato ovules caused early ovule senescence, thus demonstrating that the observed overaccumulation of *PbEIL1* in ovules was indeed related to ovule senescence. Unlike in ‘1913’, however, ovule senescence was not obvious in tomato at the early stage of development. We speculated that this latter result was due to the rapid development of ovules after fertilization, which may have weakened the effect of the ethylene signal, as developing ovules are insensitive to ethylene. A previous study has reported that ethylene signaling is activated at a suitable point in development. At this time, ethylene receptor levels are adjusted in tissues fated to die, and these tissues become appropriately sensitive to the generated ethylene signals^47^. Furthermore, ethylene does not readily cause young leaves to undergo senescence, but it can induce senescence in leaves that have reached a defined age^48^. These pieces of evidence indicate that ethylene is not the basic cause of ovule senescence but instead acts more like an executor. For example, ethylene plays an indispensable role in the senescence of degenerated ‘1913’ ovules, which are themselves the products of unsuccessful fertilization.

A recent study has demonstrated that overaccumulation of *Ein3* in synergid cells prevents pollen tube attraction, with *SAG29* activated by Ein3 playing a role in this function of synergid cells^26^. After fertilization, the Ein3-dependent ethylene response pathway is necessary for synergid cell PCD^25^. These results indicate that Ein3 participates in many early developmental processes. Because the mechanism by which Ein3 regulates ovule senescence is still unclear, we focused on the downstream genes of Ein3 in the present study. Wan et al.^33^ identified an integument-specific gene, *Cysp1*, associated with PCD in the inner integument in *B. napus*. In our current investigation, we confirmed that PbCysp1 is very closely related to BnCysp1 and that PbEIL1 can promote the expression of *PbCysp1* (Figs. 3b, 4c, d, and 5b, c). This regulatory mechanism is a novel discovery in pear and provides a reference for future research on early ovule development. In addition, our genomic sequence analysis uncovered no differences in amino acid sequences among PbACS, PbACO, PbEIL1, and PbCysp, but we found six single-nucleotide polymorphisms (SNPs) in the PbEIL1 promoter sequence of ‘1913’ that differed from those in ‘Starkrimson’ and ‘Dangshansu’ pear (Supplementary Figs. S5 and S6). An analysis of *cis*-acting elements allowed us to exclude the influences of these sites on the ethylene signal response, but other potential functions of these six SNPs need to be further studied.

**Fig. 6:**
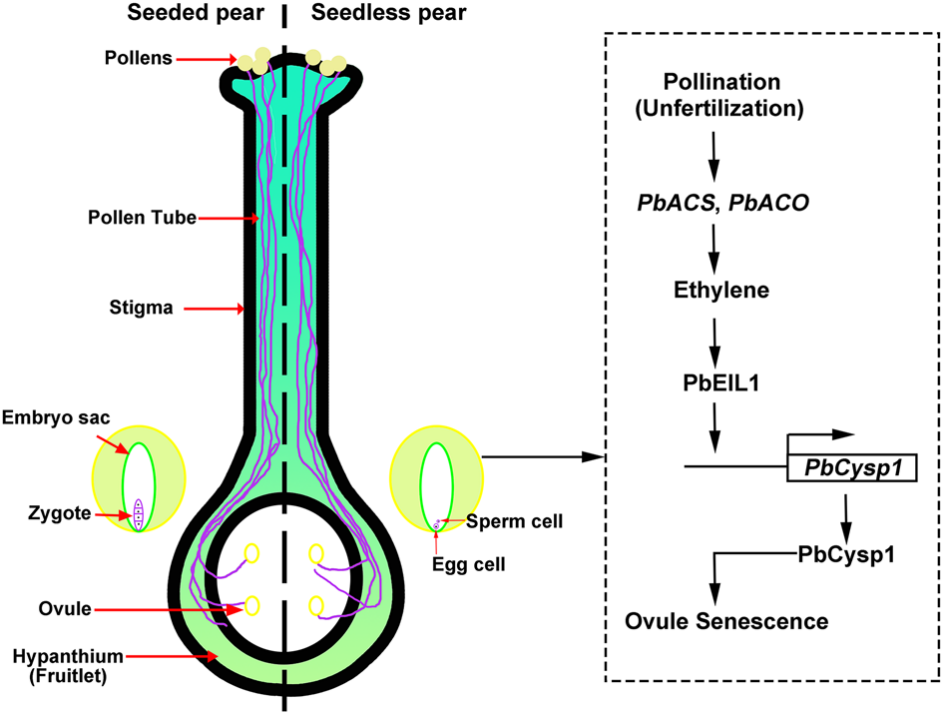
Model depicting the mechanism of ethylene involvement in unfertilized ovule abortion. Ethylene overaccumulates in the absence of fertilization, and the ethylene signal promotes *PbEIL1* expression. PbEIL1 then binds to the promoter of *PbCysp1* and enhances its action, resulting in ovule abortion

Taken together, our data indicate that pollination activates ethylene biosynthesis in pear ovules and that overaccumulation of ethylene in unfertilized ovules is involved in ovule abortion. Taking into account the transcriptomic data, the results of expression pattern analysis, and the results of interaction and activation assays on candidate genes, we conclude that ethylene signaling enhances the transcription of *PbEIL1*, after which PbEIL1 promotes the expression of *PbCysp1* for participation in unfertilized ovule senescence (Fig. 6).

## Materials and methods

### Plant materials and growth conditions

The pear samples used in this study were collected from a pear orchard in Meixian, Shaanxi, China, in 2018 and 2019. All selected materials were bagged 2 days before flowering to prevent pollination. The following materials were used: the seedless pear cultivar ‘1913’ (*Pyrus communis* L.), and the seeded pear cultivars ‘Dangshansu’ (*Pyrus bretschneideri* Rehd.) and ‘Starkrimson’ (*Pyrus communis*). The pear cultivar ‘1913’ is a hybrid selected from a cross of ‘Bartlett’ (*Pyrus communis* L.) with a male parent of ‘Zaosu’, a hybrid of ‘Pingguoli’ (*Pyrus bretschneideri*), and ‘Shenbuzhi’ (*Pyrus communis*). The seedless characteristic of ‘1913’ has proven stable over many years of observation. The sun-exposed surfaces of ‘1913’ fruits are bright red, and the fruits need to be softened after maturity, which is a well-known characteristic of European pear fruits. All samples were randomly screened to ensure uniform size and the absence of mechanical damage. Several samples were used for observation of ovules, and some samples were fixed in FAA fixative solution to observe histocytes of ovules. The remaining samples were immediately frozen in liquid nitrogen and stored at −80 °C for total RNA extraction.

Tomato (*Solanum lycopersicum* L. ‘Micro-Tom’) was used as the material for the transgenic experiment. For tomato germination and growth, the seeds were first imbibed in 50 °C water for 4–5 h and then bagged in wet gauze. After germination for 48 h, the seedlings were transferred to pots (120 × 100 mm) containing a mixture of peat:vermiculite (1:1, v/v) and cultured in a growth chamber (25 °C, relative air humidity of 70–80%, 16-h/8-h day/night photoperiod and photon flux of 115 µmol/m^2^/s). The seedlings were irrigated daily with Hoagland’s solution, and natural light was supplemented with Osram lamps (Powerstar HQI-BT, 400 W) to provide 16 h of daylight^49^.

### Transcriptome analysis

Ovules collected at 0 DAP, 3 DAP, and 7 DAP were used for RNA sequencing. The ovules were collected from ‘1913’ and ‘Dangshansu’ pear. Total RNA was extracted using an RNAprep Pure Plant Kit (Tiangen, Beijing, China) according to the manufacturer’s instructions. RNA contamination and degradation were monitored on 0.8% agarose gels, and RNA purity was detected on a Tecan MultiskanGO full-wavelength multifunctional enzyme labeling instrument (Thermo, Waltham, MA, USA). A total of 1 µg of RNA per sample was used as input material for RNA sample preparation. Sequencing libraries were prepared by random fragmentation of the cDNA sample and used for Illumina HiSeq sequencing. After constructing a reference genome index in Bowtie v2.2.3, paired-end clean reads were aligned to the reference genome using TopHat v2.0.12. The reads mapped to each gene were counted in HTSeq v0.6.1. The RNA-sequencing data from ovules at 0 DAP were used as controls. A false discovery rate <0.001 was used as the threshold for the significance of DEGs. Genes were annotated using the ‘Dangshansu’ pear database (http://www.ncbi.nlm.nih.gov/genome/?term=pyrus) as a reference. Three independent biological replications were sequenced and analyzed.

### Histological and microscopic examination

Ovules of ‘1913’ and ‘Dangshansu’ were collected, fixed in FAA for 24 h, dehydrated through an ethanol series, and embedded in paraffin wax. For safranin and fast green staining, tissues were cut into longitudinal sections (5 μm thickness), dewaxed with xylene, hydrated with an ethanol series and stained with safranin and fast green before microscopic examination (BX51 + PD72 + IX71, Olympus, Japan) and imaging. For Alcian blue staining, the tissues were prepared and sectioned in the same manner, and then the tissues were stained with Alcian blue and 1% nuclear fast red before microscopic examination and imaging^50^.

### Phytohormone analysis

Ovules collected at 7 DAP were used as the materials for phytohormone analysis. Samples (0.1 g) were ground in liquid nitrogen and placed in 2 mL centrifuge tubes. Then, 1 mL of ethyl acetate was added, and the samples were shaken at 2000 rpm for 10 min. Next, the suspensions were centrifuged at 12,000 rpm for 10 min, and the supernatant was withdrawn. The supernatant was dried under nitrogen gas, dissolved in 200 µL of methanol and filtered through a 0.22-µm filter membrane before testing. GA_1_, GA_3_, GA_4_, IAA, and ACC levels were determined by ultra-performance liquid chromatography-tandem mass spectrometry (UPLC-MS/MS) (AB SCIEX TripleTOF 5600+, Darmstadt, IN, USA). UPLC-MS/MS was performed using an ACQUITY UPLC HSS T3 (1.8 mm, Waters, USA) column (2.1 × 100 mm). The mobile phase solvent was the same as that described by Balcke et al.^51^, and the injection volume was 2 µL. The mass spectrometry conditions were as follows: a spray voltage of 4500 V and air curtain, nebulizer and auxiliary gas pressures of 15, 65, and 70 psi, respectively. The atomizing temperature was 400 °C. Each sample consisted of three replicates from independent experiments.

### ACC spraying assay

The fruit of ‘Dangshansu’ was collected at 30 DAP, and then the ovules were separated and precultured in a Petri dish with phosphate-buffered solution (PBS)-moistened filter paper at 16 °C in the dark for 12 h. The ovules with black surfaces were removed, and the rest of the ovules were treated with water (as controls) or 10 µM ACC^52^. Then, the development of seeds was observed, and the samples were stained with trypan blue to verify cell death.

### Trypan blue staining

Trypan blue staining was performed as described previously, with minor modifications^53^. For staining, the samples were immersed in boiled lactophenol (glycerol:lactic acid:liquid phenol:distilled water, 1:1:1:1) with 0.25 mg/mL trypan blue for 20 s. Next, the samples were destained with destaining buffer (ethanol:liquid phenol:lactic acid, 2:1:1) at 65 °C for 1 h and washed three times with 75% (v/v) ethanol. Then, the samples were photographed under a microscope (MZ10F, Leica, Germany).

### Quantitative real-time PCR (qRT-PCR) assay

Total RNA was extracted using an RNAprep Pure Plant Kit (Tiangen, Beijing, China). The RNA concentration and quality were assessed by UV spectrophotometry and on a 0.8% agar ethidium bromide-stained gel, respectively. Next, 1 µg of total RNA was reverse-transcribed into cDNA using a PrimeScript RT Reagent Kit with gDNA Eraser (Takara, Dalian, China). qRT-PCR amplifications were performed on an ABI instrument (Thermo Fisher Scientific, Massachusetts, USA) using a SYBR Premix Ex Taq Kit (Takara). The primers used for qRT-PCR are listed in Supplementary Table S1. Three biological replicates were used in the assay, and *PbActin7* (LOC103926846) was used as a reference gene. For data analysis, the relative expression level of each gene was calculated using the cycle threshold (Ct) 2^−ΔΔCt^ method^54^.

In this study, we collected ovules at 0 DAP, 3 DAP, and 7 DAP from ‘1913’, ‘Dangshansu’, and ‘Starkrimson’ to analyze the differential expression of candidate genes. In the tissue-specific expression assay, we collected petals, sepals, hypanthium, stamens, stigmas, and ovules at 7 DAP and leaves at 153 DAP from ‘1913’ for analysis.

### Production of transgenic tomato plants

The complete coding sequence (CDS) of *PbEIL1* was fused to the PeFBP7 promoter of a pBI121 binary vector. The PeFBP7 promoter sequence was cloned from petunia DNA. *PeFBP7* is an ovule-specific expression gene. The primers are listed in Supplementary Table S1. The *p*FBP7::PbEIL1 construct was transformed into tomato using *Agrobacterium tumefaciens* strain EHA105. For transformation, the unfolded cotyledons were first cut off before the true leaves emerged. Then, the cotyledons were cultured for 1 day in the dark and placed on wet filter paper in Petri dishes containing solidified preculture (PC) medium (MS salts supplemented with vitamins, 3% [w/v] sucrose, 100 mg/L myo-inositol, 1 mg/L kinetin, and 0.7% [w/v] agar). Next, they were immersed in bacterial suspensions (OD_600nm_ = 0.4) containing 200 µM acetosyringone for 10 min. After that, the bacterial suspension on the explants was removed with filter paper, and the explants were then cultured in the dark on PC medium for 2 days. Then, the explants were transferred to PC medium containing 2 mg/L zeatin, 300 mg/L cefotaxime, and 100 mg/L kanamycin. Explants that developed resistant calli produced shoots, which were excised and placed on rooting medium (MS salts, 2% [w/v] sucrose, 100 mg/L myo-inositol, 1 mg/L thiamine, 1 mg/L IAA, and 0.7% [w/v] agar). The rooted explants were cultured in pots containing vermiculite, watered with Hoagland’s solution, and conditioned in a growth chamber before being transferred to a greenhouse.

### GUS staining assay

For GUS staining, 20-day-old tomato fruit was stained with 5-bromo-4-chloro-3-indolyl glucuronide at 37 °C for 12 h as described by Fillatti et al.^55^.

### Homologous evolution analysis

The amino acid sequences of senescence-specific cysteine protease genes in pear, *A. thaliana* and *B. napus* were aligned using ClustalW^56^. A phylogenetic tree was constructed using MEGA 5.10 with the neighbor-joining statistical method. In addition, 1000 bootstrap replications were performed to test the phylogeny.

The genes included were as follows: PbSAG101 (*Pyrus bretschneideri*, LOC103928442), PbSAG101-like (*Pyrus bretschneideri*, LOC103928444), PbSAG101-like (*Pyrus bretschneideri*, LOC103939636), PbSAG101-like (*Pyrus bretschneideri*, LOC103939637), PbZingipain2-like (*Pyrus bretschneideri*, LOC103944110), PbSAG39-like (*Pyrus bretschneideri*, LOC103945424), PbCysp1 (*Pyrus bretschneideri*, LOC103928267), BnCysp1 (*Brassica napus*, AF448505), and ATSAG12 (*Arabidopsis thaliana*, AT5G45890).

### Yeast one-hybrid assay

A yeast one-hybrid assay was performed following the manufacturer’s instructions for a Matchmaker Gold Yeast One-Hybrid System Kit (Clontech, Mountain View, CA, USA). The full-length CDS of *PbEIL1* was amplified and inserted into the MCS of pGADT7 AD, and the approximately 500 bp promoter of *PbCysp1* was inserted into pAbAi bait vectors. The pAbAi bait vectors were linearized and transformed into Y1HGold separately. Then, the colonies were selected on a plate without uracil for selective glucose synthesis. By colony PCR analysis (Matchmaker Insert Check PCR Mix 1; Clontech), we confirmed the correct integration of the plasmids into the genome of Y1HGold. Then, the bait yeast strains were cultured on SD/–Ura medium with different concentrations of aureobasidin A (AbA) to select the minimum inhibitory concentration. Next, the AD-prey vectors were transformed into the bait yeast strains and selected on an SD/-Leu/AbA plate. All transformations and screenings were performed three times. The related primers are listed in Supplementary Table S1.

### Dual-luciferase assay

The promoter sequence of *PbCysp1* cloned from ‘1913’ genomic DNA using PrimeSTAR Max Premix (Takara) was inserted into the dual-LUC plasmid pGreenII 0800-LUC (reporter vector). The full-length CDS of *PbEIL1* was cloned into the MCS region of a pGreenII 0029 62-SK binary vector (effector vector)^57^. The effector and reporter vector constructs are shown in Fig. 5c.

Each recombinant plasmid was transferred into *Agrobacterium* strain GV3101. *Agrobacterium* cells containing PbEIL1-62sk and pPbCysp1-LUC were mixed at a 1:1 ratio before infiltration into 4-week-old *Nicotiana benthamiana* leaves. The controls received mixed injections of *Agrobacterium* cells with an empty 62sk vector and PbCysp1-LUC, and the control and treatment plants were injected on the left and right sides of the same leaf. Then, the injected plants were grown in darkness at room temperature for 1 day and in light for 2 days. Next, the leaves were collected in PBS for the dual-luciferase assay. The ratio of firefly luciferase enzyme activity to Renilla luciferase enzyme activity was analyzed using a dual-luciferase reporter assay system (Promega, Madison, WI, USA) on a Tecan Infinite M200 PRO full-wavelength multifunctional enzyme labeling instrument (Tecan, Hombrechtikon, Switzerland). Five independent biological replicates were analyzed. The related primers are listed in Supplementary Table S1.

### Statistical analysis

The data were subjected to analysis of variance and tested for significant (^*^P < 0.05, ^**^P < 0.01) treatment differences using Duncan’s test and Student’s *t*-test. The results are presented as mean ± standard deviation (SDs) of three replicate samples.

## Supplementary information

Table S3 DEGs related to hormone synthesis and signal transduction pathways.

Supplemental figures.

Table S1 List of primers.

Table S2 KEGG enrichment analysis of DEGs.
