# Supplementary material for: PbEIL1 acts upstream of PbCysp1 to regulate ovule senescence in seedless pear
Source: Hortic Res. 2021 Mar 10;8:59. doi: 10.1038/s41438-021-00491-5 (PMC7943805; doi:10.1038/s41438-021-00491-5)

**PbEIL1 acts upstream of *PbCysp1* to regulate ovule senescence in seedless pear**

**Running title:** Ethylene involvement in ovule senescence

**Huibin Wang ^1, 2^, Haiqi Zhang ^1, 2^, Fangfang Liang ^1^, Liu Cong ^1^, Linyan Song ^1^, Xieyu Li ^1^, Rui Zhai ^1^, Chengquan Yang ^1^, Zhigang Wang ^1,*^, Fengwang Ma ^1^, Lingfei Xu ^1, *^**

**^1^** College of Horticulture, Northwest A&F University, Taicheng Road NO.3, Yangling, Shaanxi Province, China.

**^2^** The authors have equal contributions to this study.

*** Correspondence:** Zhigang Wang ([wzhg001@163.com](mailto:wzhg001@163.com)) or Lingfei Xu (Email: [lingfxu2013@sina.com](mailto:lingfxu2013@sina.com) Tel.: +86-29-87081023; fax: +86-29-87082613.).

H. B. Wang ([wanghuibin069@163.com](mailto:wanghuibin069@163.com));

H. Q. Zhang ([1440473827@qq.com](mailto:1440473827@qq.com));

F. F. Liang ([liangfangfang@nwafu.edu.cn](mailto:liangfangfang@nwafu.edu.cn));

L. Cong ([2335487375@qq.com](mailto:2335487375@qq.com));

L. Y. Song ([linyans@yeah.net](mailto:linyans@yeah.net));

X. Y. Li ([lixieyu@qq.com](mailto:lixieyu@qq.com));

R. Zhai ([zhongdishaonian@sina.com](mailto:zhongdishaonian@sina.com));

C. Q. Yang ([cqyang@nwsuaf.edu.cn](mailto:cqyang@nwsuaf.edu.cn));

F. W. Ma ([fwm64@nwsuaf.edu.cn](mailto:fwm64@nwsuaf.edu.cn) ).


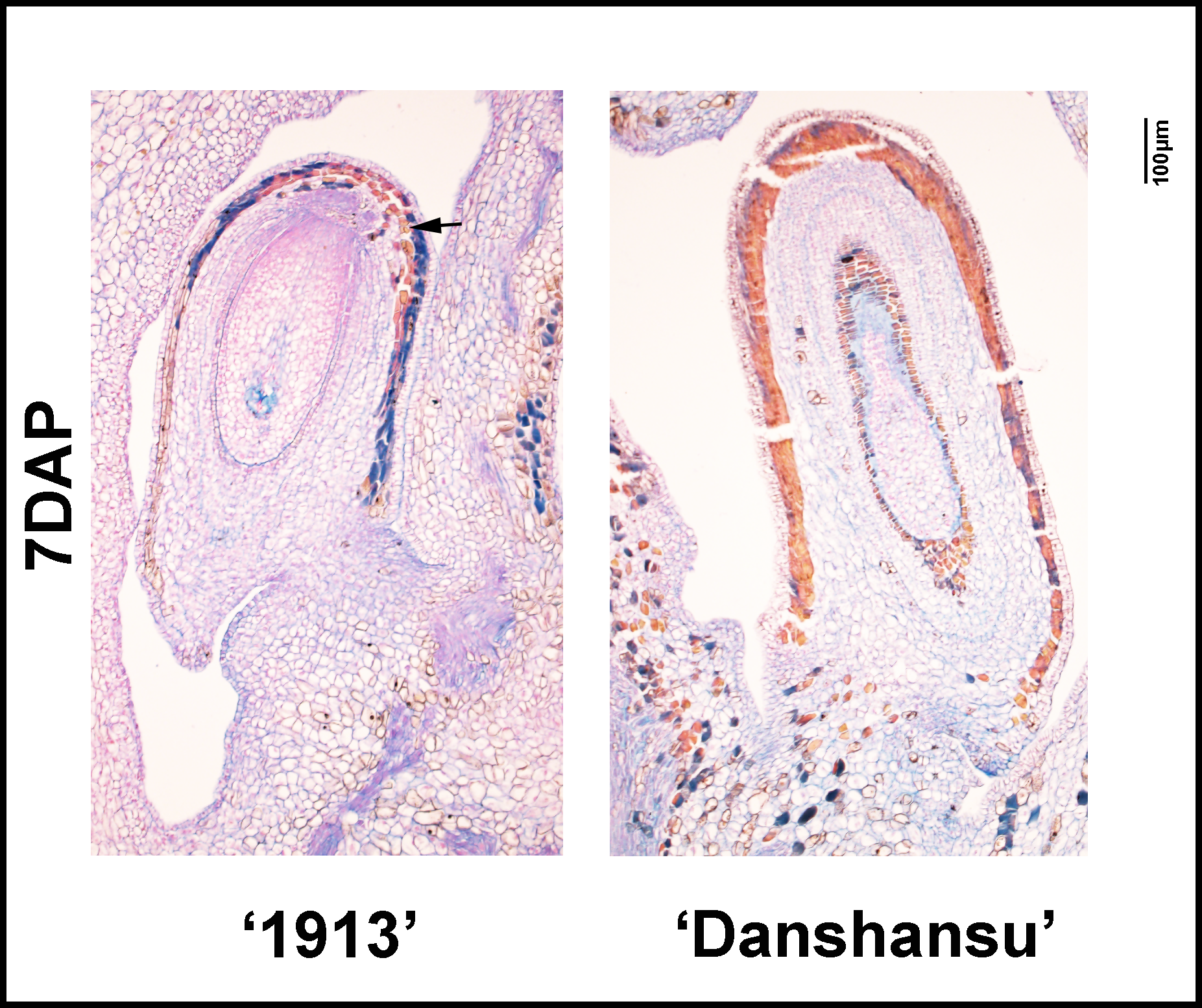


**Fig. S1** **Observation of ovule development in ‘1913’ and ‘Dangshansu’.** The observation of ovule development that stain with alcian blue and nuclear fast red at 7DAP. The black arrows indicate the area of the integument. DAP, days after pollination.


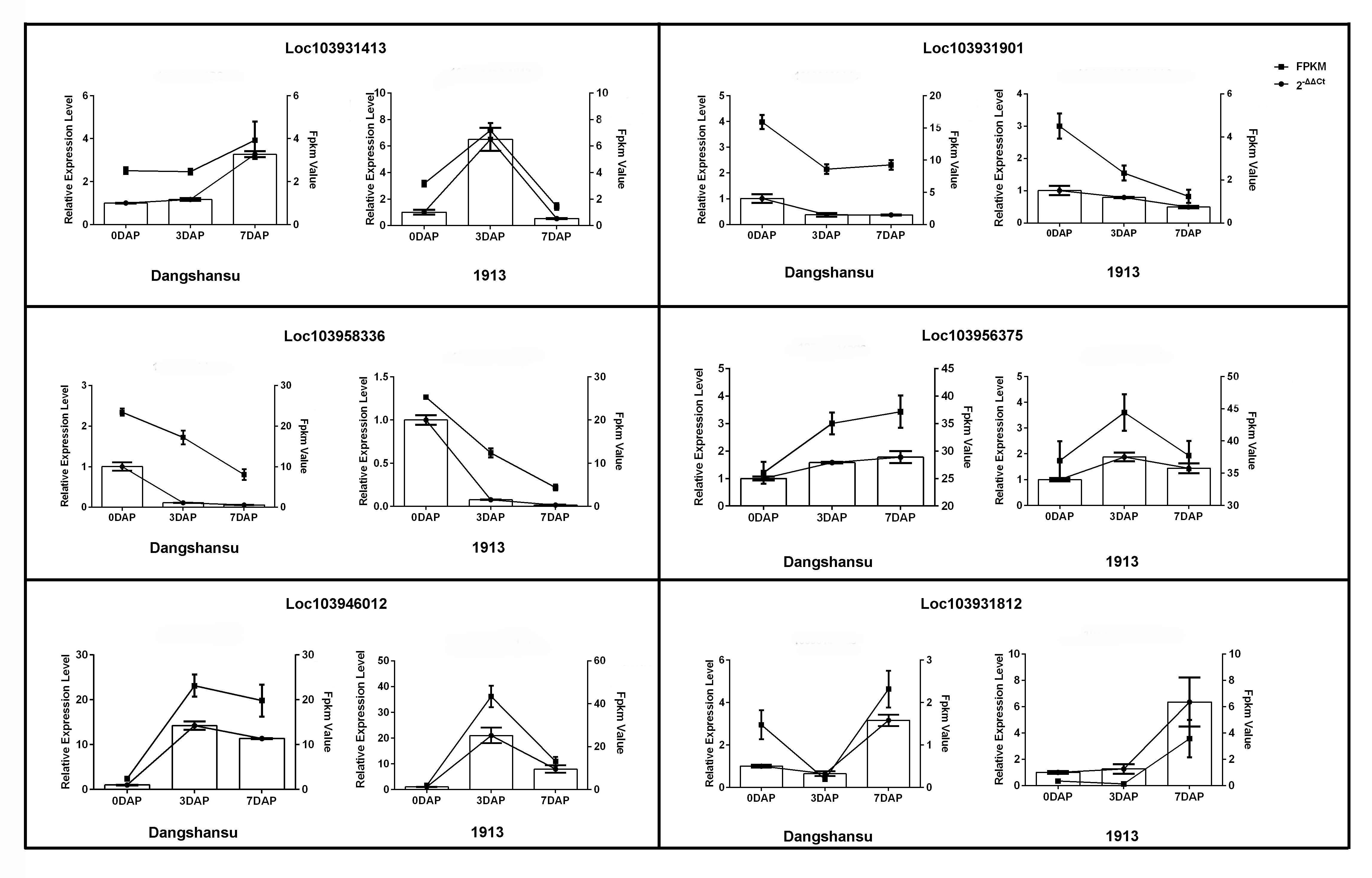


**Fig. S2** **The reliability analyze of transcriptome data by qRT-PCR assay.** Error bars represent the standard deviation of three independent PCR mixtures. DAP, days after pollination.


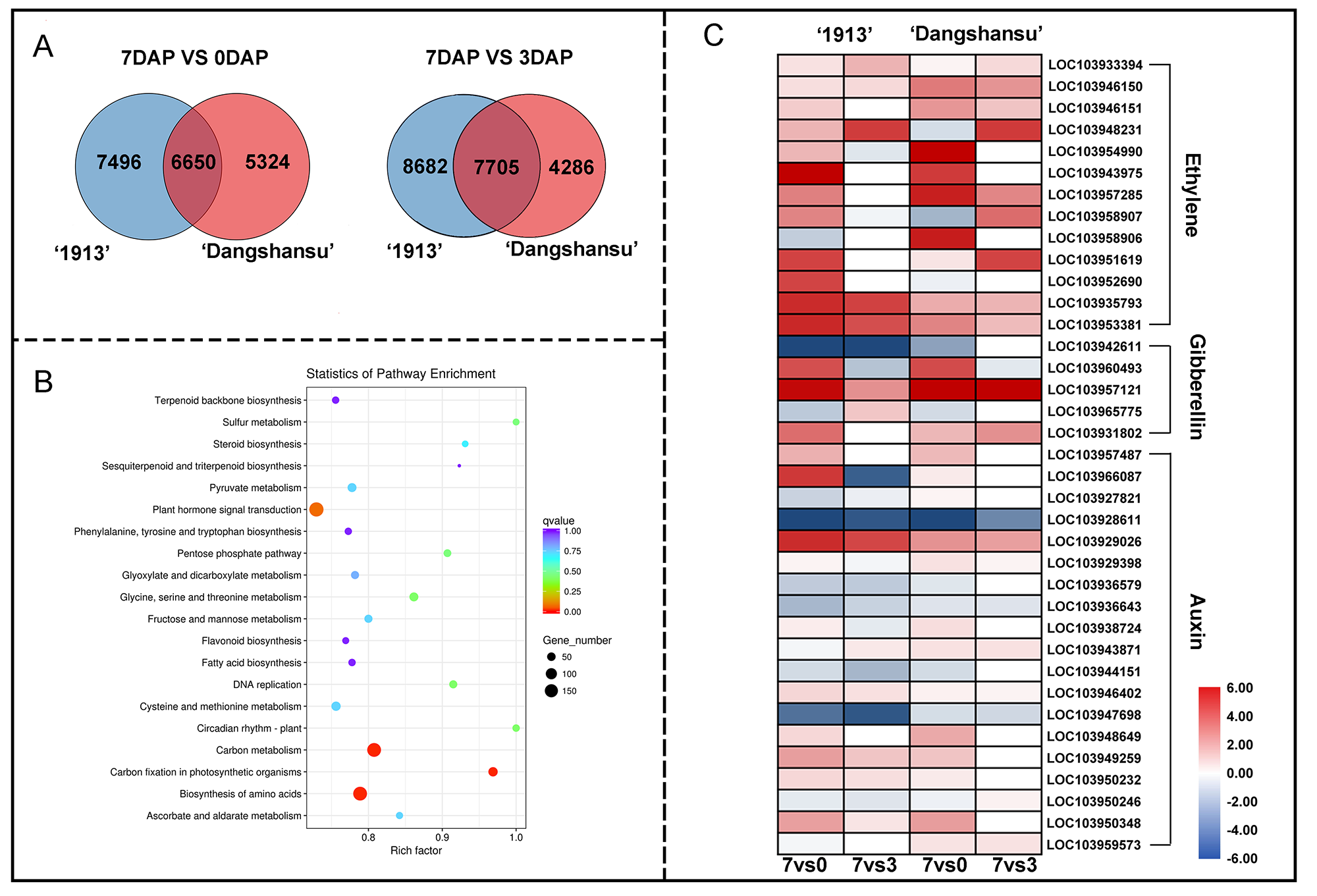


**Fig. S3** **RNA-Seq analysis of the ‘1913’ and ‘Dangshansu’ ovules at 0DAP and 3DAP, compared with the ovule at 7DAP. a** Venn diagram of the number of different expression genes (DEGs) between ‘1913’ and ‘Dangshansu’ ovule at 0 ,3 and 7DAP. **b** The Kyoto Encyclopedia of Genes and Genomes (KEGG) enrichment analysis of 11350 DEGs. **c** Heatmap analyzed the DEGs of the hormone-related genes in ‘1913’ and ‘Dangshansu’ at 0, 3 and 7DAP. DAP, days after pollination.


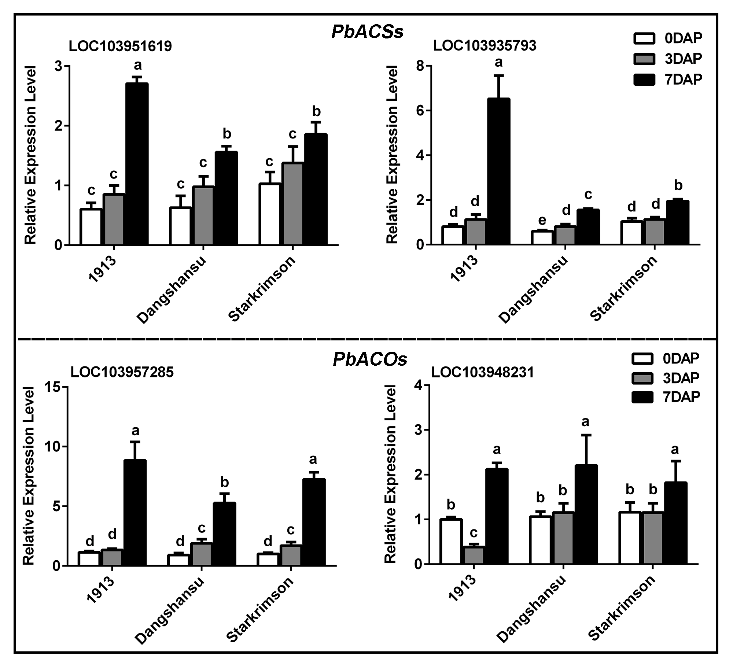


**Fig. S4** The expression patterns of the other *PbACSs* and *PbACOs* at different stages in seeded cultivars and ‘1913’. The expression levels of each gene in ‘Starkrimson’ at 0DAP were normalized as 1.0. Data are the means ± SDs of three biological replicates. Different letters between bars indicate significant differences at *P* < 0.05 (Duncan's range test). DAP, days after pollination.


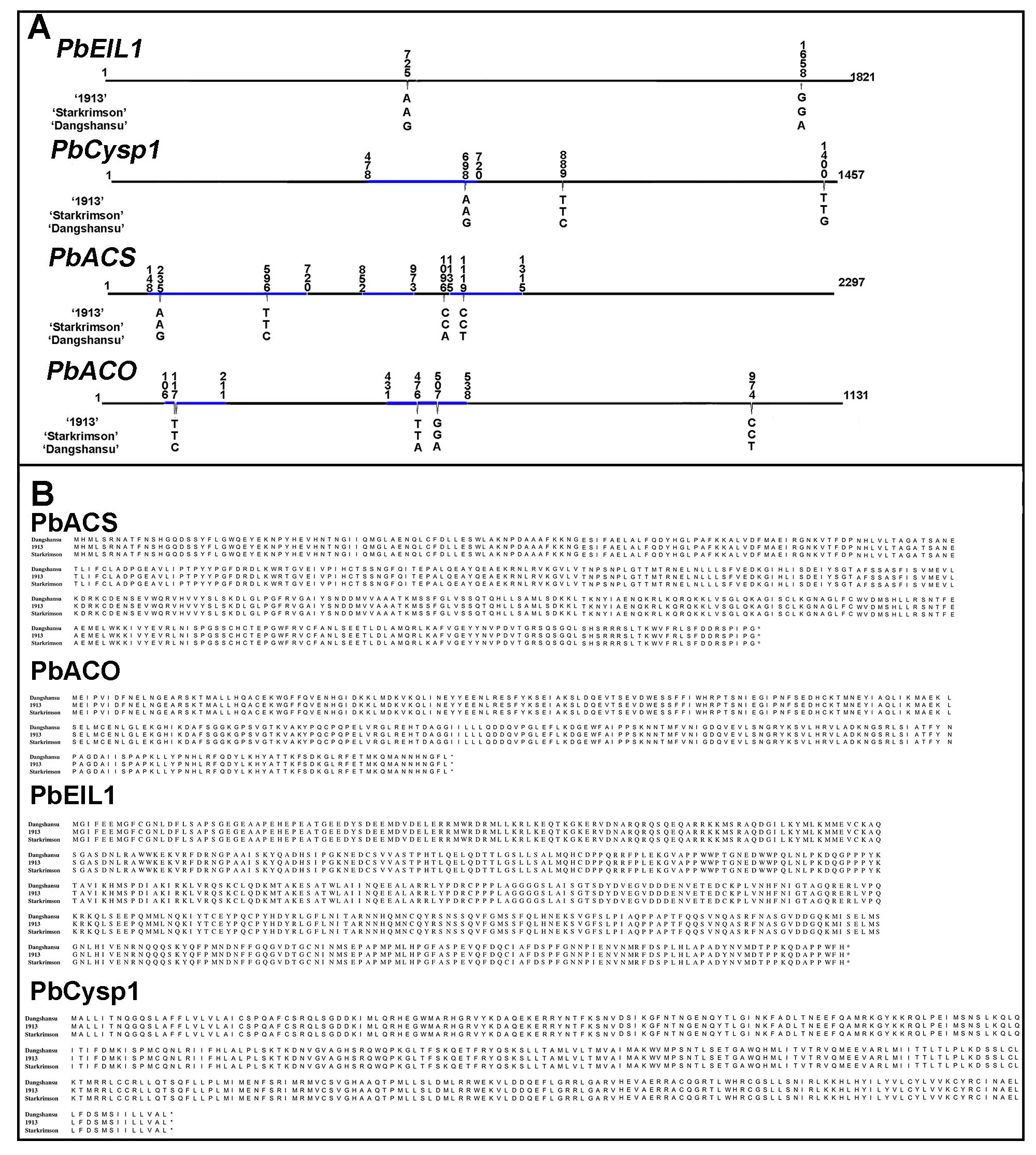


**Fig. S5** Analyzing the nucleotide and amino acid sequences of *PbACS*, *PbACO*, *PbEIL1* and *PbCysp1* in ‘Dangshansu’, ‘1913’ and ‘Starkrimson’. a Nucleotide sequences analysis of candidate genes., and the black lines refer to the exon sequence and the blue lines refer to the intron sequence. b Amino acid sequence analysis of candidate genes.


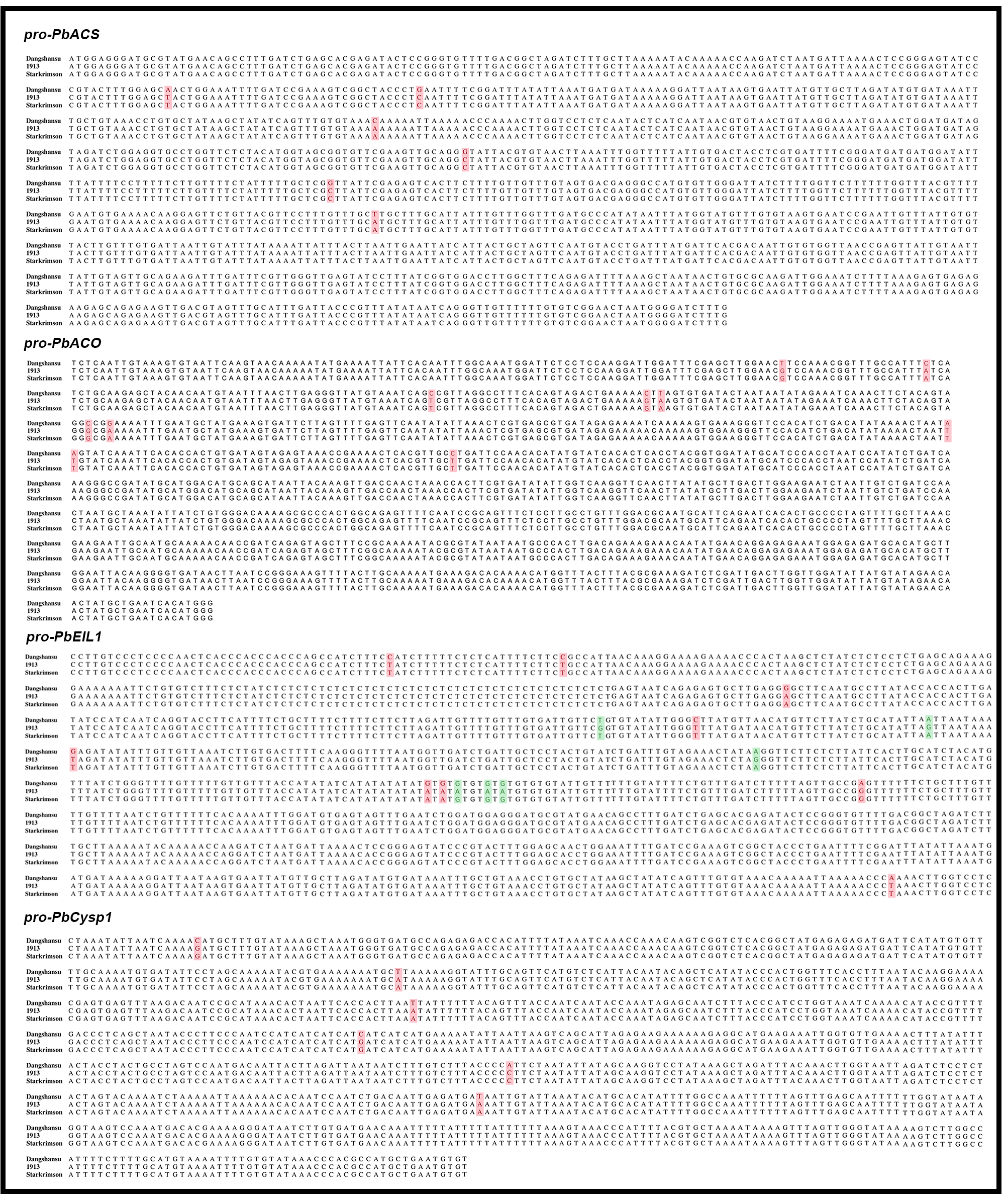


**Fig. S6** Analyzing the promoter sequences of *PbACS*, *PbACO*, *PbEIL1* and *PbCysp1* in ‘Dangshansu’, ‘1913’ and ‘Starkrimson’. Each sequence is about 1000bp. The pink markers mean that the site in ‘1913’ is different from that in one of the two varieties, and the green marker means that the site in ‘1913’ is completely different from the other two varieties.


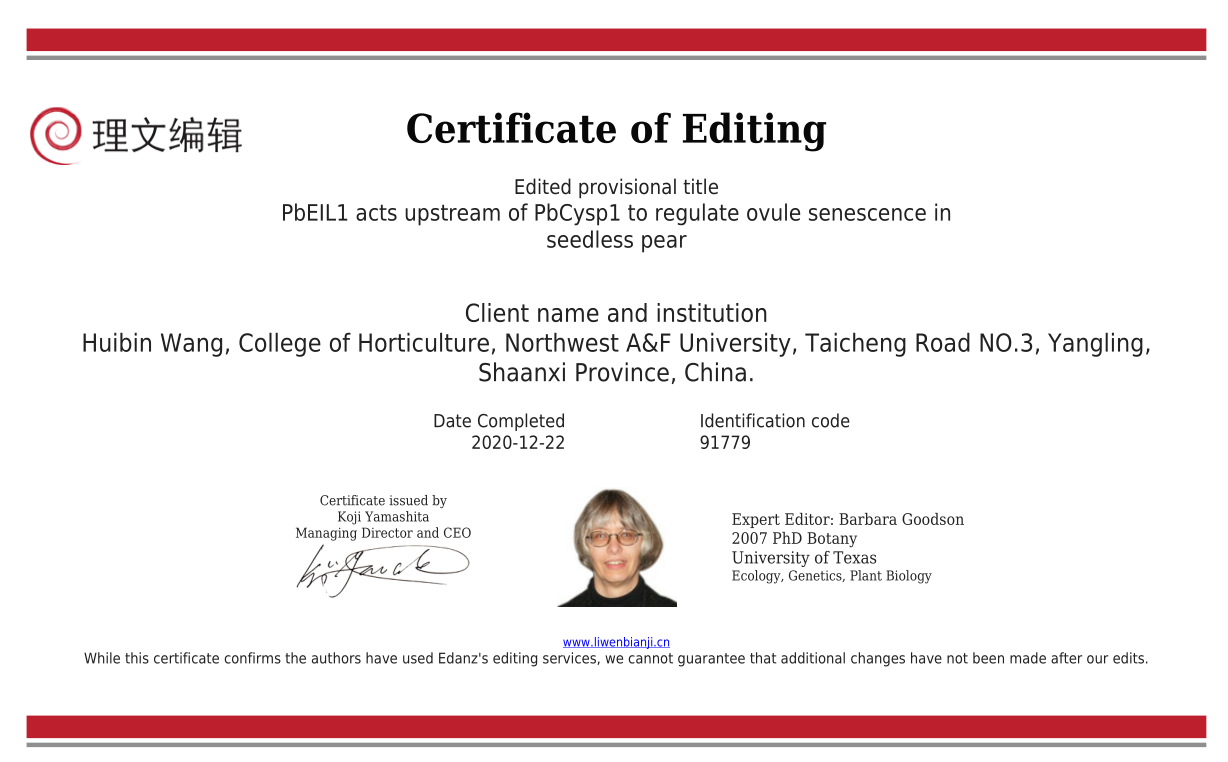

Supplement: Supplementary file 2 — Supplemental figures. [file 41438_2021_491_MOESM2_ESM.docx]
